# Developmental and Genetic Considerations in a Neonate With Pectus Excavatum, Bilateral Hydroceles, Indirect Inguinal Hernia, and Type 2 Ileal Atresia

**DOI:** 10.7759/cureus.78350

**Published:** 2025-02-01

**Authors:** Natalie Nagib, Daniela Avila, Abby L Denham, Pedro Montanez

**Affiliations:** 1 Medicine, Lake Erie College of Osteopathic Medicine, Bradenton, USA; 2 Pediatrics, AdventHealth Sebring, Sebring, USA

**Keywords:** congenital hydrocele, ilial atresia, indirect inguinal hernia, neonatology, pectus excavatum, pediatric surgery

## Abstract

This case study describes a combination of congenital anomalies observed in a full-term male neonate, including pectus excavatum, bilateral hydroceles, an indirect inguinal hernia, and type 2 ileal atresia. The neonate, delivered by cesarean section at 39 weeks and one day, presented with pectus excavatum and bilateral hydroceles, confirmed both prenatally and postnatally. Postpartum, the neonate experienced feeding difficulties and failed to pass meconium within 24 hours of birth. Diagnostic imaging revealed dilated bowel loops consistent with intestinal obstruction, necessitating surgical intervention for ileal atresia. At a four-month follow-up, the patient was found to have developed an inguinal hernia. This constellation of anomalies has not been previously documented, highlighting the importance of comprehensive evaluation, multidisciplinary care, and patient counseling in such cases. Furthermore, this report underscores the need for further investigation into potential genetic syndromes, developmental disruptions in mesodermal-derived structures, or in utero vascular insults that may underlie the concurrent presentation of these anomalies.

## Introduction

Congenital anomalies often occur in isolation, but their cooccurrence may reveal shared embryological, genetic, or environmental etiologies. This report describes a rare combination of anomalies-pectus excavatum (PE), bilateral hydroceles, and type 2 ileal atresia (IA)-in a neonate. PE, commonly known as "funnel chest," represents approximately 95% of congenital chest wall anomalies [1]. It is characterized by a depression in the anterior chest wall, primarily involving the third to seventh costocartilages or ribs, with the most pronounced indentation typically located near the xiphisternum [1]. PE occurs in approximately one in 300 to one in 1,000 live births and is more prevalent in male patients, with a 5:1 male-to-female ratio [1].

A hydrocele is an accumulation of fluid within the tunica vaginalis of the testis [2]. Congenital hydrocele results from the failure of the processus vaginalis to obliterate, maintaining a connection between the peritoneal cavity and the scrotum [2]. Hydroceles can also be structurally classified as either communicating or non-communicating [2]. A communicating hydrocele is characterized by a patent processus vaginalis, allowing fluid to flow between the peritoneal cavity and the scrotum, whereas a non-communicating hydrocele involves fluid accumulation within the scrotum without an open connection to the peritoneal cavity [2]. The primary communicating hydrocele is the most common type seen in pediatric patients, with 80%-90% of term male infants born with a patent processus vaginalis [2]. Hydroceles are more commonly found on the right side, with left-sided hydroceles occurring in 25% of cases and bilateral hydroceles in 15% [2].

Inguinal hernias are classified as either congenital or acquired and further subdivided into direct and indirect types. Indirect hernias, which may be congenital or acquired, result from weakness in the inguinal canal that allows the intestine to protrude through the internal and external inguinal rings, sometimes descending into the scrotum [3]. Direct hernias, which are always acquired, are more common in middle-aged and older men [3]. They occur within Hesselbach's triangle, medial to the lower epigastric vessels, and may extend along the inguinal canal to the scrotum, although they lie outside the spermatic cord [3]. Inguinal hernias are more frequently observed on the right side (75%), attributed to the delayed descent and closure of the right testicle [4]. Approximately one-third of cases present before six months of age, with bilaterality occurring in 15%-20% of children [4]. Among infants, bilateral hernias are observed in about 10% of full-term cases and nearly 50% of those born prematurely or with low birth weight [4]. In pediatric cases, indirect hernias are the most common type, nearly always resulting from incomplete closure of the processus vaginalis [5].

IA, a prevalent cause of neonatal intestinal obstruction, occurs in approximately one in 5,000 to one in 14,000 live births [6]. This condition may present as single or multiple lesions, with distal lesions generally manifesting later than proximal ones [6]. IA is sometimes associated with other anomalies, including cardiac defects, gastroschisis, and cystic fibrosis [6]. Fewer than 10% of IA cases involve extra-abdominal abnormalities, likely due to the late occurrence of vascular compromise during pregnancy [6]. There is no established connection between IA and parental health conditions, and chromosomal abnormalities are identified in less than 1% of cases [6].

The Grosfeld classification system categorizes intestinal atresia into four types, each with distinct anatomical features and varying frequencies. Type 1, involving an internal membrane with serosal continuity and no mesenteric defect, accounts for approximately 14% of jejuno-ileal atresia cases [6,7]. Type 2, characterized by blind proximal and distal pouches connected by a fibrous cord with serosal discontinuity, also represents about 14% of cases [6,7]. Type 3a, defined by serosal discontinuity with a V-shaped mesenteric defect, constitutes approximately 16% of cases [6,7]. Type 3b, known as "apple peel" atresia, involves proximal jejunal atresia with a short ileal segment coiled around the ileocolic artery and accounts for about 9% of cases [6,7]. Finally, type 4, characterized by multiple atresias, comprises approximately 10% of cases [6,7]. These frequency estimates are derived from a study analyzing 63 cases of jejuno-ileal atresia [6,7]. These conditions are each relatively well documented in the literature, but their simultaneous presentation has not been previously reported, raising questions about potential underlying syndromic or developmental links.

## Case presentation

**Figure 1:**
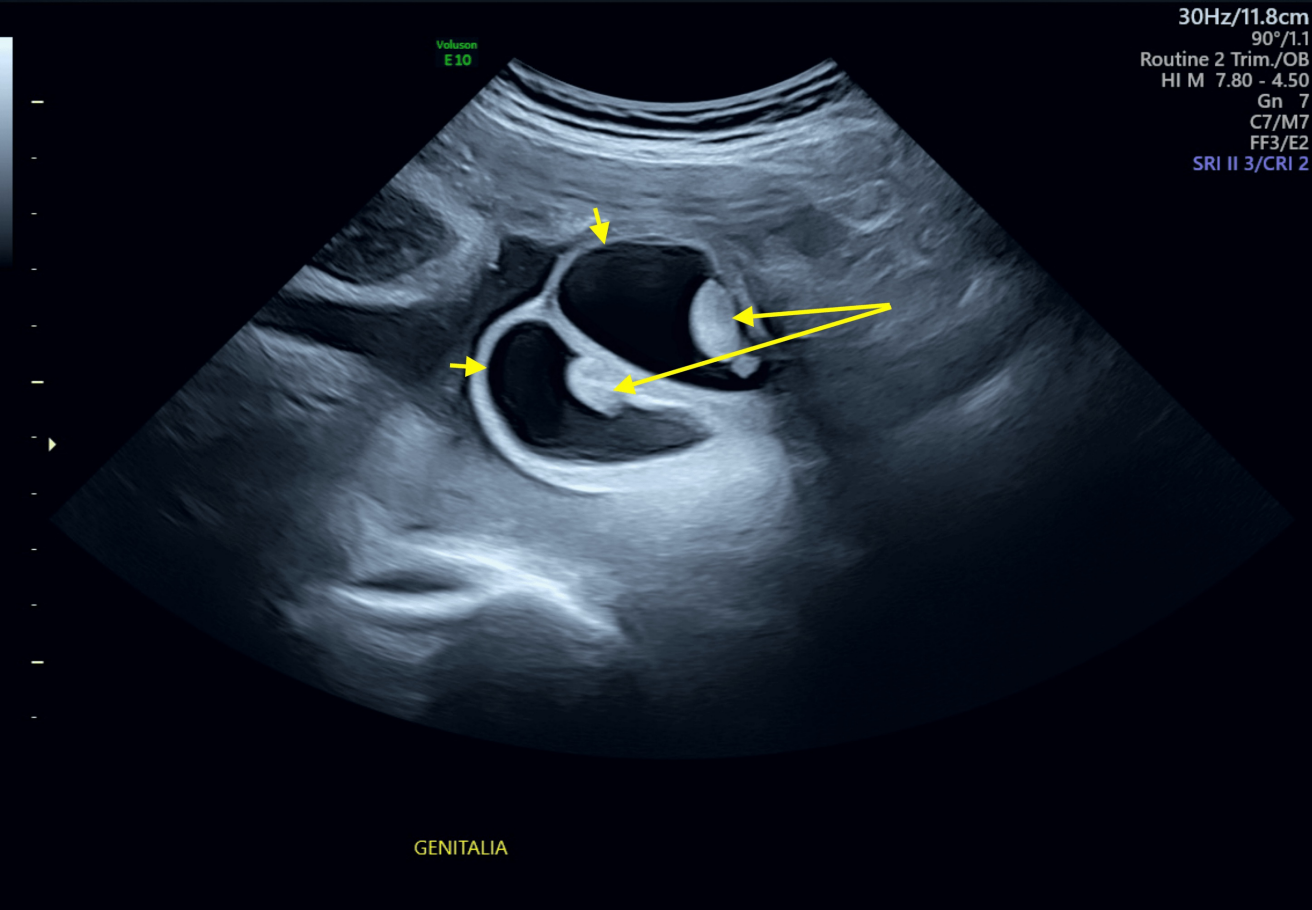
Transvaginal growth US demonstrating bilateral hydrocele. Scrotal fluid collection compatible with hydrocele (short arrows), surrounding testes (long arrows).

A male neonate was delivered via scheduled cesarean section at 39 weeks and one day of gestation without complications. The neonate was admitted for evaluation due to an inability to pass meconium and presented with feeding difficulties. The mother, aged 30, had a medical history notable for bundle branch block and PE, which was also observed in the patient upon examination. Family history revealed that the patient’s maternal grandmother had a similar condition of PE. Bilateral hydroceles were noted at birth and confirmed during a growth ultrasound performed at 36 weeks and one day of gestation (Figure 1).

On examination, the infant had a birth weight of 3,030 g (25th percentile) and a length of 49.5 cm (43rd percentile). Initial vital signs were stable, and the appearance, pulse, grimace response, activity, and respiration (APGAR) scores-each criterion scored from 0 to 2-were 9 at one minute and 9 at five minutes. The APGAR score provides a quick assessment of a newborn’s health at one and five minutes postdelivery, with a total score of 7-10 considered normal. Physical examination revealed bilateral hydroceles and PE but was otherwise unremarkable. However, on the first day of life, the infant failed to pass meconium. Rectal stimulation was performed, yielding no stool, which raised concern for possible bowel obstruction. The infant had two episodes of bilious vomiting but remained non-distressed, with no signs of abdominal distention.

**Figure 2:**
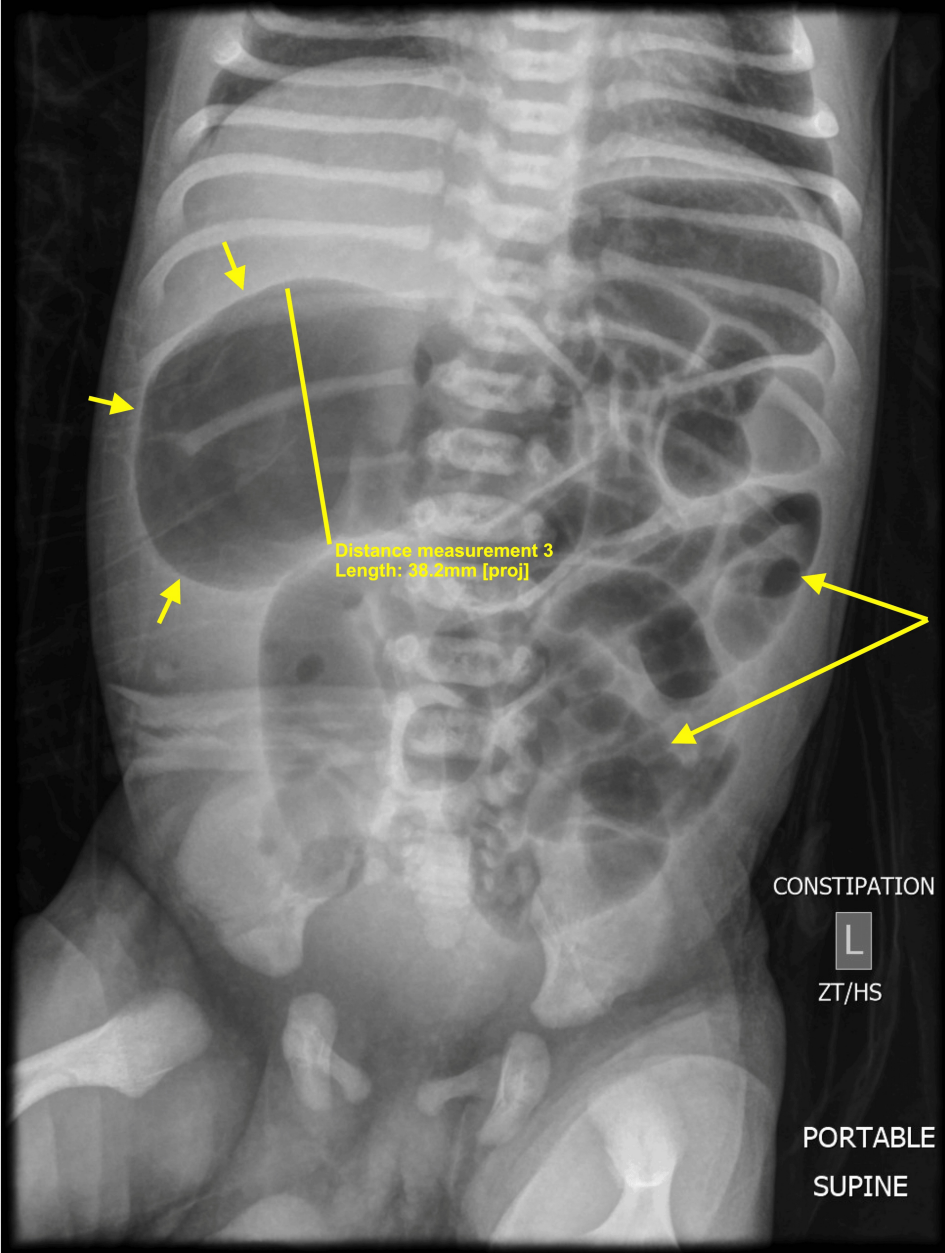
Abdominal radiograph shows significant gaseous distention (small arrows) and dilatation of colonic loops with prominence of small bowel gaseous distention (long arrows).

On the second day of life, an abdominal X-ray showed significant gaseous distention measuring up to 4.4 cm in the right upper abdomen (Figure 2).

**Figure 3:**
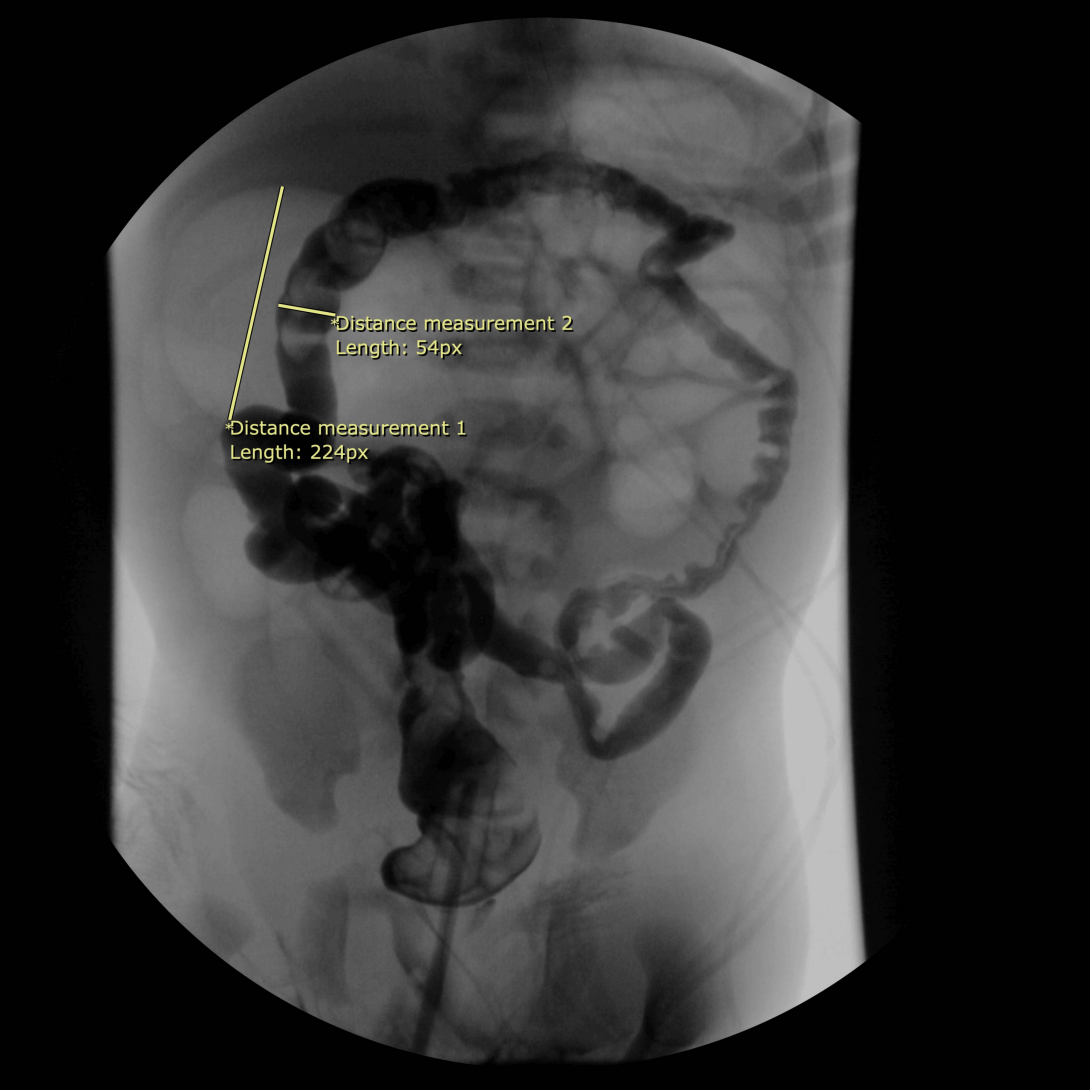
Barium enema depicting a very dilated loop of bowel is noted in the right upper quadrant, and the appearances are consistent with small bowel atresia possibly in the distal jejunum or proximal ileum.

Additionally, there was the prominence of small bowel gaseous distention in the left abdomen, alongside a paucity of bowel gas in the pelvis. No free air was detected beneath the diaphragm, and no significant accumulation of stool was observed. These findings raised concerns about possible IA or other causes of dynamic distal colonic obstruction. A barium enema performed later that day indicated that the rectal tube passed freely from the rectum throughout the large bowel into the cecum, with reflux into the distal small bowel (Figure 3).

Based on the clinical findings and imaging results, the patient required surgery, and following the procedure and biopsy, a diagnosis of type 2 IA was confirmed. Initial management included NPO (nothing by mouth) status and intravenous fluids, initiated due to abdominal distension. The patient underwent exploratory laparotomy on the second day of life, which led to the resection of the atretic segment and a primary ileoileal anastomosis.

**Figure 4:**
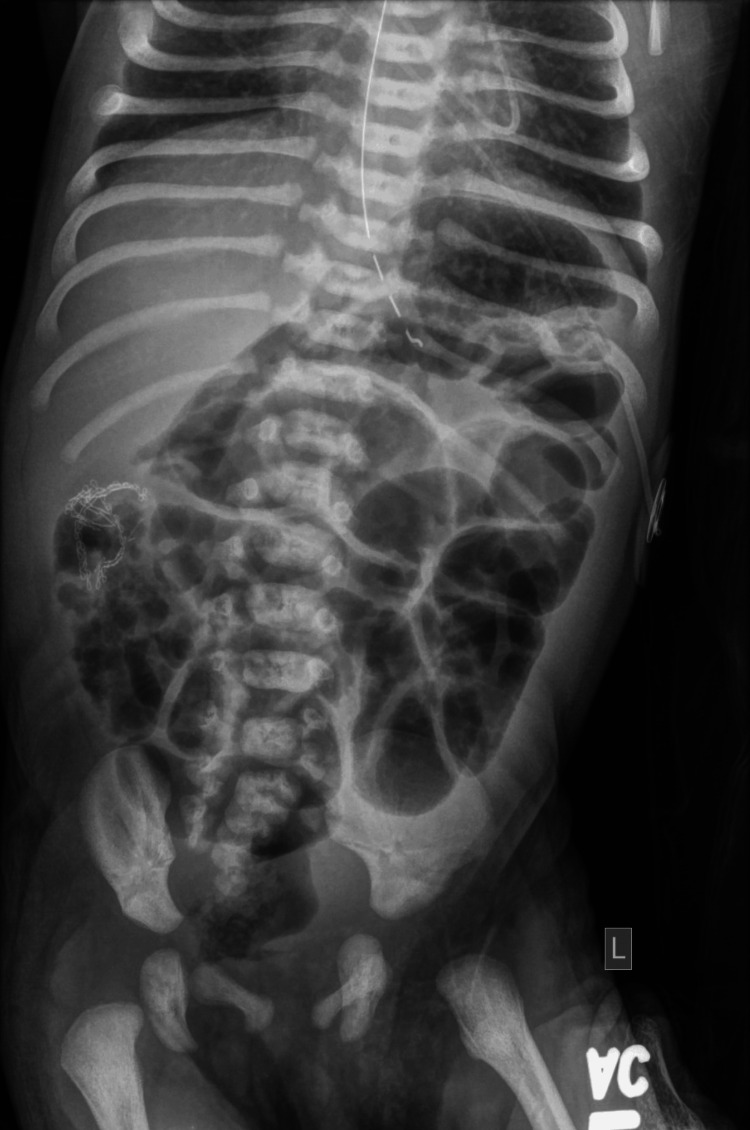
Abdominal radiograph shows the status post-surgical repair of ileal atresia with a relatively normal bowel gas pattern, which is now non-obstructive.

On day eight of life, a follow-up abdominal X-ray demonstrated a non-obstructive bowel gas pattern following the surgical repair of IA (Figure 4).

The imaging showed an enteric tube in the stomach, with no free air or other abnormal findings in the intra-abdominal organs. Surgical stapler clips from the recent abdominal surgery were visible.

Post-surgery, the patient remained stable, with gradual advancement of feeding. He was discharged on day 15 of life, having tolerated oral feeds and established regular stooling. Follow-up appointments were scheduled with pediatric surgery and general pediatrics.

At the four-month follow-up with the pediatric surgeon, the patient was evaluated for bilateral hydroceles. Physical examination revealed a left-sided hydrocele extending to the cord, without evidence of communication with the abdomen. A possible hernia and a smaller hydrocele were noted on the right side. No inguinal hernias were observed on the left. Based on these findings, the surgeon recommended continued observation, with plans for reassessment at one year of age to monitor for any changes requiring intervention. Long-term follow-up will monitor hydrocele resolution, potential hernia development, and any related structural concerns. Coordination among pediatric surgery, pediatric gastroenterology, and general pediatrics was essential, with effective communication between hospitals ensuring comprehensive care.

## Discussion

The combination of conditions-PE, bilateral hydroceles, indirect inguinal hernia, and type 2 IA-in a single neonate has not been identified in the literature. While isolated cases of each condition are relatively common, the simultaneous occurrence of all four, coupled with the absence of similar reports, likely qualifies this as a rare clinical scenario. Furthermore, genetic testing may uncover a unified explanation for this constellation of anomalies. This could be attributed to a shared germ cell lineage, a connective tissue etiology, or in utero environmental factors.

PE, hydrocele, and inguinal hernias are congenital abnormalities that arise from disruptions in the mesodermal layer during early embryogenesis [8]. The coexistence of these conditions in this patient suggests the possibility of a shared underlying etiology. Although these abnormalities involve distinct anatomical systems, there are documented cases of chest wall deformities cooccurring with genitourinary anomalies [9,10]. While this specific combination of defects has not been previously reported, evidence exists of defined syndromes that encompass features observed in this patient, such as Shprintzen-Goldberg, Aarskog-Scott, and Noonan syndromes [9]. While none of the syndromes listed fully account for this patient’s presentation, they highlight the existence of other syndromes that combine two or more of these potentially linked features.

In addition to an embryological connection, this patient presents with defects suggestive of a connective tissue-based anomaly. Both PE and hydrocele/inguinal hernias are considered connective tissue disorders, often linked to abnormalities in collagen, elastic fibers, and extracellular matrix components [9]. In this case, the patient exhibits a non-communicating hydrocele on the left side and a communicating hydrocele on the right, along with a right-sided indirect inguinal hernia [4]. Indirect inguinal hernias, congenital in origin, result from the persistence of a patent processus vaginalis, in contrast to acquired direct inguinal hernias [8].

Marfan syndrome and Ehlers-Danlos syndrome (EDS) are well-documented connective tissue disorders associated with a range of systemic abnormalities. In Marfan syndrome, hallmark features include PE and, less commonly, urogenital anomalies such as hydroceles [11]. While there is a less direct association between Marfan syndrome and type 2 IA, the syndrome belongs to the broader spectrum of connective tissue disorders, which could potentially impact the intestines as well [11]. Similarly, subsets of EDS frequently exhibit PE, and while direct associations with bilateral hydroceles or IA are rare, the connective tissue laxity characteristic of EDS can contribute to urogenital abnormalities, including hydroceles [12].

Environmental factors including maternal nutrition, infections, and mechanical factors play a significant role in the development of congenital conditions such as bilateral hydroceles, PE, and type 2 IA. These factors often interact with genetic predispositions, influencing embryonic development and contributing to anatomical anomalies. Poor maternal nutrition or metabolic imbalances during pregnancy can disrupt lymphatic and vascular development [13], which may lead to fluid accumulation in the scrotal area, while compromised vascular development can interfere with drainage and contribute to hydrocele formation. Furthermore, maternal nutritional deficiencies, including inadequate intake of vitamins and minerals like folic acid, have been associated with an increased risk of various congenital anomalies. Proper nutrition is essential for the development of cartilage and bone structures during pregnancy [13].

Nutritional deficiencies can impair placental function and alter the uterine environment, leading to inadequate oxygen and nutrient delivery to the fetus, which may disrupt normal gastrointestinal development [13]. The pathogenesis of IA is thought to stem from an intrauterine vascular accident affecting the mesenteric vessel branches in the midgut, resulting in ischemic necrosis of the fetal bowel [6]. Other causes of in utero vascular disruption, such as intussusception, volvulus, and gastroschisis, have also been associated with IA [6]. Factors like thromboembolic occlusions, vasoconstrictive drugs, and maternal smoking during the first trimester have been shown to increase the risk of intestinal atresia [6]. While not documented in this patient’s history, it is impossible to rule out environmental factors such as maternal nutritional status as potential contributors to the observed anomalies.

Maternal infections during pregnancy, such as intrauterine infections, also have the potential to alter embryonic development. These infections can affect the growth of the urogenital system, sometimes resulting in hydroceles as a complication [14]. Additionally, intrauterine factors such as adhesions or constrictions can mechanically interfere with the developmental processes of the intestines [6]. Furthermore, these mechanical and developmental disruptions increase the risk of ischemic events, which can compromise bowel integrity during crucial periods of fetal development [6]. Mechanical factors during fetal development, such as external pressures from conditions like oligohydramnios, adhesions, or intrauterine constraint, may also contribute to chest wall deformities [15]. These mechanical influences can affect the positioning and growth of thoracic structures [15]. Although specific mechanical influences are not explicit in this case, it is worth considering and noting that such factors remain possible contributors to the observed anomalies.

The pathogenesis of IA results in ischemic necrosis of the fetal bowel, constituting a medical emergency that requires immediate surgical resection of the atretic segment [6]. In contrast, conditions such as PE, hydrocele, and inguinal hernia typically manifest their adverse consequences later in life [1-4]. For instance, PE-associated cardiopulmonary complications usually emerge or worsen during the pubertal growth spurt, rather than in the newborn or infant stages [1]. Monitoring of PE progression involves pulmonary function tests (PFTs), cardiac imaging, and electrocardiograms (EKGs) [1]. Similarly, hydroceles and inguinal hernias often resolve spontaneously within the first year of life and are generally managed conservatively during infancy [2-4]. For this patient, a follow-up in one year is planned to assess whether any intervention will be necessary.

Although no definitive evidence yet indicates how treatment plans might differ for a patient with such a presentation, this case contributes valuable data for future researchers investigating potential links between congenital syndromes. Retrospective analyses of cases like this may help identify patterns, refine diagnostic criteria, and develop tailored management strategies, ultimately guiding the creation of standardized approaches for evaluating and treating similar presentations. Additionally, it has been suggested that the diagnostic challenge posed by the subtlety of representative clinical signs in genetic disorders necessitates a standardized approach for patients with PE. Many representative clinical signs are not likely to be linked to genetic disorders because of the rarity of genetic disorders associated with PE [9,10]. These obstacles make the decision to refer a patient presenting with PE for genetic analysis often difficult, mandating a standardized approach [9,10].

We propose that the patient’s congenital anomalies may be linked to an underlying syndrome, highlighting the need for a comprehensive evaluation that includes genetic testing. Such a workup could help anticipate additional disorders that might influence surgical or treatment plans. For example, another study suggests that if a patient with PE is diagnosed with an underlying heritable connective tissue disorder, surgical repair should be delayed until skeletal development is complete [16]. The familial occurrence of PE, noted in the patient, mother, and maternal grandmother, raises questions about a possible genetic link influencing the severity of the condition across generations. For instance, if the mother required surgical intervention, it is worth exploring whether this increases the likelihood of the baby needing similar treatment in the future. Furthermore, should an inheritable risk be identified, reproductive counseling for both the mother and the child is crucial to ensure they are well informed about the risks and empowered to make sound decisions regarding their health and reproduction.

## Conclusions

This case underscores the complexity of managing multiple congenital anomalies in a single patient, emphasizing the importance of a multidisciplinary approach to understanding and treating such presentations. The concurrent presentation of type 2 IA, PE, bilateral congenital hydroceles, and indirect inguinal hernia represents a clinical question that has not been previously documented in the literature. While each condition is relatively well understood individually, their simultaneous occurrence raises new questions about potential shared embryological, genetic, or environmental factors. The familial pattern of PE, observed in both the patient, mother, and maternal grandmother, further suggests a possible hereditary component that may influence the severity and clinical progression of these anomalies. This case also highlights the crucial role of prenatal and early postnatal imaging in the identification and management of such conditions. As the patient continues to develop, careful monitoring will be essential to determine whether additional interventions will be necessary for the management of PE, hydroceles, and indirect inguinal hernia. Ultimately, this case points to the need for further research into the interplay between these congenital anomalies and the genetic or environmental factors that may contribute to their cooccurrence. A better understanding of these underlying mechanisms could lead to improved management strategies and preventative measures for similar cases in the future.

## References

[1] Sharma G, Carter YM (2024). Pectus excavatum. StatPearls [Internet].

[2] Huzaifa M, Moreno MA (2024). Hydrocele. StatPearls [Internet].

[3] Kasalovic M, Gojko I, Aleksandar J, Nikola M, Milica M (2023). Inguinal herniation associated with hydrocele: inguinal herniation associated with hydrocele. J Surg Med.

[4] Lao OB, Fitzgibbons RJ Jr, Cusick RA (2012). Pediatric inguinal hernias, hydroceles, and undescended testicles. Surg Clin North Am.

[5] Brandt ML (2008). Pediatric hernias. Surg Clin North Am.

[6] Osuchukwu OO, Rentea RM (2024). Ileal atresia. StatPearls [Internet].

[7] Burjonrappa S, Crete E, Bouchard S (2011). Comparative outcomes in intestinal atresia: a clinical outcome and pathophysiology analysis. Pediatr Surg Int.

[8] Somuncu S, Somuncu ÖS (2021). A comprehensive review: molecular and genetic background of indirect inguinal hernias. Visc Med.

[9] Billar RJ, Manoubi W, Kant SG, Wijnen RM, Demirdas S, Schnater JM (2021). Association between pectus excavatum and congenital genetic disorders: a systematic review and practical guide for the treating physician. J Pediatr Surg.

[10] Billar R, Heyman S, Kant S, Wijnen R, Sleutels F, Demirdas S, Schnater JM (2024). Early-onset pectus excavatum is more likely to be part of a genetic variation. Eur J Pediatr Surg.

[11] Arena F, Impellizzeri P, Antonuccio P, Montalto S, Racchiusa S, Romeo C (2009). Neonatal intrathoracic gastric volvulus in Marfan's syndrome. Minerva Pediatr.

[12] Wong M, Javid PJ (2020). Bilateral femoral hernias in a male child as the initial presentation of Ehlers-Danlos syndrome. J Pediatr Surg Case Rep.

[13] Ricci TA, Boonpattrawong N, Laher I, Devlin AM (2023). Maternal nutrition and effects on offspring vascular function. Pflugers Arch.

[14] Fujita H, Yoshii A, Maeda J, Kosaki K, Shishido S, Nakai H, Awazu M (2004). Genitourinary anomaly in congenital varicella syndrome: case report and review. Pediatr Nephrol.

[15] David VL (2022). Current concepts in the etiology and pathogenesis of pectus excavatum in humans-a systematic review. J Clin Med.

[16] Tocchioni F, Ghionzoli M, Messineo A, Romagnoli P (2013). Pectus excavatum and heritable disorders of the connective tissue. Pediatr Rep.

